# Genetic susceptibility to Lewy body disease and the distribution of Lewy pathology

**DOI:** 10.64898/2026.10.21.26366146

**Published:** 2026-10-24

**Authors:** Lesley Y. Wu, Jared W. Taylor, Miko Valori, Tatiana Georgiades, Tessa du Toit, Ana-Luisa Gil-Martinez, Eleanor J. Stafford, Kristin Levine, Zih-Hua Fang, Cornelis Blauwendraat, Andrew B. Singleton, Sonja W. Scholz, Bryan J. Traynor, John Hardy, Patrick Chinnery, Richard Cain, Claire Troakes, Djordje Gveric, Catriona McLean, Seth Love, Andrew King, Andrew C. Robinson, Federico Roncaroli, Claire Shepherd, Glenda Halliday, Laura Parkkinen, Christopher M Morris, Johannes Attems, Colin Smith, Geidy E. Serrano, Thomas G. Beach, Steve Gentleman, Thomas T. Warner, Tammaryn Lashley, Zane Jaunmuktane, Owen A. Ross, Dennis W. Dickson, Lasse Pihlstrøm, Raquel Real, Huw R Morris, the Global Parkinson’s Genetics Program (GP2)

## Abstract

Genome-wide association studies of Parkinson’s disease and dementia with Lewy bodies largely rely on clinical diagnoses, which can misclassify cases and cannot characterise the anatomical distribution of Lewy pathology. Autopsy cohorts allow identification of variants associated both with developing Lewy body disease and with the neocortical extent of its pathology.

We meta-analysed genome-wide data from 2,661 individuals of European ancestry with autopsy-confirmed Lewy body disease and 12,068 controls (2,124 autopsy-confirmed) from the Global Parkinson’s Genetics Program (GP2) and the National Institutes of Health NeuroBioBank, and estimated genetic correlation with clinically diagnosed Alzheimer’s disease. In 2,309 cases from the GP2 and the Mayo Clinic Jacksonville Brain Bank, we compared neocortical (n = 1,362) with brainstem or limbic (n = 947) Lewy pathology, adjusting for Alzheimer’s disease co-pathology, age at death and sex.

Under a neuropathological case definition, five established loci reached genome-wide significance: *APOE* ε4 (OR 2.27, 95% CI 2.06-2.50), *GBA1* E326K (OR 2.57, 95% CI 2.01-3.28), *SNCA* (OR 1.35, 95% CI 1.23-1.48; *TMEM175* p.M393T (OR 1.30, 95% CI 1.19-1.43), and *HLA* class II (OR 0.69, 95% CI 0.61-0.79). Lead associations differed by antemortem diagnosis. Across all cases, the *SNCA* association was indexed by a 5′ signal previously associated with dementia with Lewy bodies and rapid eye movement sleep behaviour disorder, rather than the canonical 3′ Parkinson’s disease variant. Among 1,216 cases diagnosed during life with Parkinson’s disease, with or without dementia, the 3′ variant was the lead *SNCA* signal, and the *APOE* ε4 effect was attenuated (OR 1.57, 95% CI 1.37-1.81). Genetic correlation with Alzheimer’s disease (0.33, *P* = 0.02) persisted after excluding the *APOE* region (0.31, *P* = 0.03). Within cases, *APOE* ε4 was the only susceptibility locus associated with neocortical involvement (OR 1.70, 95% CI 1.45-1.98; P = 2.5 × 10 ^−1^ ^1^ ), including among 666 GP2 cases without significant Alzheimer’s disease co-pathology (OR 1.76, 95% CI 1.18-2.63; P = 0.005). Each additional ε4 allele was also associated with a more advanced Lewy pathology stage (OR 1.76, 95% CI 1.50-2.06).

Autopsy-confirmed Lewy body disease shares its major susceptibility loci with clinically defined disease. Of these, only APOE ε4 was also associated with neocortical involvement, independently of measured Alzheimer’s disease co-pathology, suggesting that it influences not only whether Lewy pathology develops but how far it extends.

